# The COVID-19 Stress Perceived on Social Distance and Gender-Based Implications

**DOI:** 10.3389/fpsyg.2022.846097

**Published:** 2022-05-09

**Authors:** Paolo Taurisano, Tiziana Lanciano, Federica Alfeo, Francesca Bisceglie, Alessia Monaco, Filomena Leonela Sbordone, Chiara Abbatantuono, Silvia Costadura, Jolanda Losole, Gennaro Ruggiero, Santa Iachini, Luigi Vimercati, Angelo Vacca, Maria Fara De Caro, Antonietta Curci

**Affiliations:** ^1^Department of Basic Medical Science, Neuroscience and Sense Organs, University of Bari “Aldo Moro,” Bari, Italy; ^2^Department of Education, Psychology, Communication, University of Bari “Aldo Moro,” Bari, Italy; ^3^Department of Psychology, Second University of Naples, Caserta, Italy; ^4^Department of Interdisciplinary Medicine, Occupational Health Division, University of Bari “Aldo Moro,” Bari, Italy; ^5^Department of Biomedical Sciences and Human Oncology, University of Bari “Aldo Moro,” Bari, Italy

**Keywords:** gender, depression, post-traumatic symptoms, interpersonal distance, psychological implications

## Abstract

The COVID-19 pandemic is an unprecedented event entailing long-term consequences on population health and welfare. Those who contracted the coronavirus may have suffered from both physical and mental health issues that unfold the need for tailored intervention strategies. Hence, our study aims to investigate the psychological and social consequences of COVID-19 on a sample of 86 participants, encompassing 43 patients (clinical group; 25 women; mean age = 50.4 ± 10.1 years) recruited from Bari University Hospital, 19 of whom were hospitalized due to the disease. The remaining 43 were individuals not fallen ill with COVID-19 to date (control group; 25 women; mean age = 50.4 ± 10.1 years). The investigation yielded significant gender differences in post-traumatic stress symptoms, depression, and representation of interpersonal distance (IPD), evaluated through the IES-R, the BDI-II, and the IVAS task, respectively. This pattern of results was not replicated in the control group. In general, participants who reported having experienced the most intense post-traumatic symptoms also presented a greater mood deflection and, more specifically, within the clinical group women obtained the highest scores on both scales. Women reported higher IES-R and BDI-II scores compared to men, that could indicate that women who have contracted COVID-19 are more exposed to post-traumatic and depressive symptoms. Our results also showed a significant effect of COVID-19 on IPD with a tendency of disease-experienced individuals to increase their preferred IPD from adults, children, and elderly people. Regarding gender differences in mood and proxemic behavior, a correlation between depressive symptoms and probable PTSD and a further correlation between probable PTSD and greater IPD were found in women from both clinical and control group. Overall, these findings might contribute to a better understanding of gender-based implications of the current pandemic on mental health, also leading to the development of integrated yet personalized intervention strategies.

## Introduction

On March 20, 2020, Italy was second only to China for confirmed COVID-19 cases, and the increasing number of patients in need of medical care and hospitalization led to an overload on the national healthcare system (Saglietto et al., 2020). The infection ascribed to the novel coronavirus (provisionally named 2019-nCoV) was first identified in Wuhan, China, (Huang et al., 2020), and has been designated as a severe acute respiratory syndrome (SARS-CoV-2) (Xu et al., 2020). As the disease may be contracted through aerosol particles and droplets inhaled at short to medium distances (Bahl et al., 2020; Morawska and Milton, 2020), several health devices, notably non-pharmaceutical interventions, have been implemented globally to reduce the transmission of the virus and “flatten the curve” of infections. Among all containment measures and preventive strategies, the maintenance of physical distance has emerged as one of the most effective (Yang et al., 2020).

The contraction of COVID-19 appears to be prevalent in the elderly, in people affected by multiple underlying conditions, and in patients presenting atypical symptoms (e.g., delirium, falls, gastrointestinal symptoms) (Vena et al., 2020; Lozano-Montoya et al., 2021). In particular, older age, co-occurrent cardiovascular diseases, and increased C-reactive protein (CRP) levels are associated with higher risks for all-cause in-hospital mortality (Vena et al., 2020). Overall, in- and out-of-hospital mortality due to the infection requires clinical awareness as post-acute sequelae and complications are relatively frequent over 12 months after hospital discharge (Mainous et al., 2021; Sykes et al., 2021; Vincent et al., 2021).

Regarding mental health consequences, the spread of COVID-19 has contributed to increased vulnerability in anxiety, fear, uncertainty, and internal unrest (Hussain, 2020; Mazza M. G. et al., 2020), entailing a significant psychological strain on the general population (Lanciano et al., 2020; Marazziti et al., 2020; Saladino et al., 2020; Amendola et al., 2021). In particular, gender was found to be among the highest predictors of the onset of depressive symptoms associated with the COVID-19 outbreak, severe lockdown, and quarantine in women (Delmastro and Zamariola, 2020). These findings appear consistent with those derived from a survey of Italians’ short-term psychological responses to the pandemic experience, which reported a higher prevalence of distress and depression among women (Hodes and Epperson, 2019; Mazza C. et al., 2020; Yan et al., 2021). It is indeed evident that COVID-19 peritraumatic processes may manifest differently based on gender and other variables such as, for instance, employment stability and dimensions of home environment (Bonati et al., 2021). Another key psychological factor potentially affecting the subjective experience of COVID-19 could be hospitalization, as COVID-19 suspected individuals that were admitted to hospital departments and clinics reported higher psychological distress and poorer quality of life after discharge (Vlake et al., 2021). In-patients might also exhibit negative emotions (e.g., fear, denial, anger, health-related concerns) during the early stage of the disease that precedes the acceptance of the diagnosis (Sun et al., 2021).

In view of the high infectious load and relevant implications associated with COVID-19 (including, among all, mortality, and morbidity), the current pandemic has also resulted in particularly poor mental health outcomes in frontline healthcare workers (Kang et al., 2020; Lai et al., 2020; Rossi et al., 2020; Tan et al., 2020). In Italy, healthcare providers showed psychological symptoms of distress compatible with depression, anxiety, and post-traumatic stress disorder (PTSD) (Di Tella et al., 2020; Putri et al., 2021). Notably, PTSD symptoms in March 2020 reached a prevalence of 29.5%, contributing to the labeling of COVID-19 as a traumatic event (Forte et al., 2020). Individuals who had been hospitalized after having contracted the virus have shown features of anxiety and depression (Kong et al., 2020), as well as symptoms consistent with PTSD before being discharged (Bo et al., 2021). Symptomatology compatible with PTSD may persist even after recovery and regardless of the direct experience of hospitalization (Einvik et al., 2021).

The relative unpredictability of health outcomes, the diversity in perceived risks of contagion, and the modes of transmission of the virus causing COVID-19 may have also changed the readiness of individuals while approaching others. Indeed, Iachini et al. (2021) showed that anxiety related to risk perception during the pandemic could affect interpersonal-space boundaries and the fear of contact with others. This readiness to approach others may be conditioned by the use of personal protective equipment (PPE), such as face masks, leading to the perception of others as safer and more reliable (Cartaud et al., 2020).

People especially worry about being infected in places with high public traffic (e.g., public transport; shops; restaurants), and within overcrowded contexts, women seem to be more concerned about COVID-19 transmission than men (Gerhold, 2020). Despite men’s greater vulnerability to encountering more serious physical consequences from COVID-19, women are more likely to disclose negative emotions and expectations about their chances of contracting the disease and experiencing its adverse effects (Alsharawy et al., 2021). Consistently, women, compared to men, report greater perception and fear regarding the severity of COVID-19 and thus tend to be more compliant with pandemic prevention measures, such as cleaning surfaces and engaging in physical distancing (Galasso et al., 2020; Alsharawy et al., 2021).

### Aim and Hypotheses

Considering the psychological burden entailed by the current pandemic, this study aims to investigate the extent to which having contracted COVID-19 may lead to changes in mood and perceived distress, and how these changes may be reflected in the perception of interpersonal distance. More specifically, the current work seeks to investigate potential sex differences that may emerge in parameters related to depression, likelihood of PTSD, and proxemic preferences toward children, adults, and the elderly. Consistent with the aforementioned literature, we expect that women could report higher scores of distress and psychological symptomatology associated with the psychosocial phenomenon of COVID-19. Indeed, the current pandemic represents a complex psychosocial phenomenon that entails, besides physical suffering, mental health implications that may also lead to behavioral changes such as a different perception of interpersonal distance. Moreover, we assume that women who contracted COVID-19 may have an even greater risk of suffering from distress and psychological symptoms. We also expect that variables such as gender and the direct experience of illness may affect self-reported proximity or distance behaviors.

## Materials and Methods

### Study Design

The study adopted a 2 × 2 design with Diagnosis (Clinical vs. Control) and Gender (Women vs. Men) as between-subjects factors. The dependent variables were depression, post-traumatic symptoms, and interpersonal distance (IPD).

### Participants

The study was conducted with the voluntary participation of 86 individuals, 43 of whom contracted COVID-19 (i.e., clinical group). The clinical group consisted of 24 subjects who were not hospitalized, including 21 individuals who suffered from a non-severe form of the disease, and 3 who contracted COVID-19 in a severe form. Within the same group of COVID-19 patients, 19 subjects have, instead, undergone hospitalization, presenting in nine cases a non-severe form of the disease, whereas the remaining 10 developed severe symptoms. Conversely, the 43 participants forming the control group reported not having contracted the disease themselves.

The subjects included within the clinical group have been enlisted at the A.O.U. Policlinico of Bari (Italy) and recruited from both the Clinic of Pneumology of the Department of Internal Medicine and the Clinics of the Department of Occupational Medicine. The survey focused on the assessment of the symptoms of post-traumatic stress, depression, and perception of interpersonal distance (IPD) through the administration of the Impact of Event Scale—Revised (IES-R) (Weiss and Marmar, 1996), the Beck Depression Inventory—Second Edition (BDI-II) (Beck et al., 1996), and a digitalized version of the Interpersonal Visual Analog Scale (IVAS) (Iachini et al., 2021), respectively.

All subjects who entered the study provided written informed consent to the evaluation and underwent the administration of the research protocol (i.e., IES-R, BDI-II, and IVAS).

### Measures

The Beck Depression Inventory—Second Edition (BDI-II) (Beck et al., 1996) is a brief and time-effective self-report tool with a cut-off of 16 and a split in severity. The inventory consists of 21 items and is rated along a 4-point Likert scale (i.e., from 0 to 3). The Italian version of the instrument (Ghisi et al., 2006) represents a reliable and valid measure of depressive disorders in the Italian context (Sica and Ghisi, 2007), allowing for the assessment of the presence and magnitude of depressive symptoms in adults over a 2-week period.

The psychological impact of stressful events has been measured using a version of the Impact of Event Scale-Revised (IES-R) (Weiss and Marmar, 1996). The IES-R is a self-report questionnaire consisting of 22 items and three sub-scales that measure medium avoidance, intrusion, and hyperarousal. Each item can be evaluated from 0 to 4 and is referred to the last seven days prior to the administration. The total score can range from 0 to 88, with higher scores representing a greater psychological impact triggered by the stressful event considered. In the present study, trauma-related stress symptomatology was assessed with specific regard to the COVID-19 event. To this end, the Italian version of the questionnaire (Giannantonio, 2005) was adopted to detect the presence of symptoms related to a likely PTSD is estimated on a cut-off basis (IES score of 33 or more).

**FIGURE 1:**
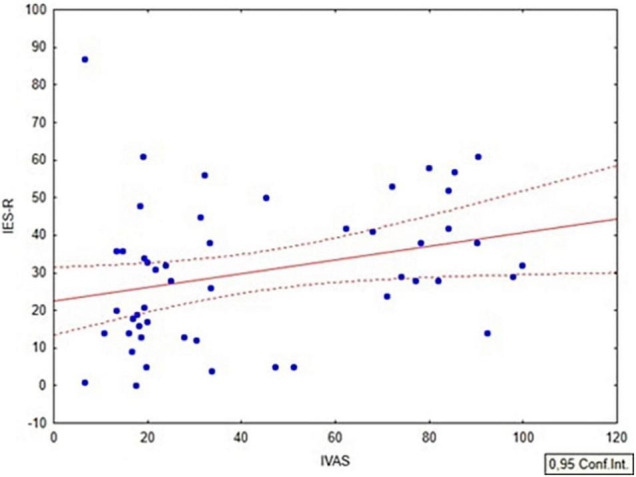
Example of the trials of IVAS. The label “TU” indicates a women participant and the other character on the opposite of the line represents the confederate from which a preferred distance must be indicated.

The software package Psytoolkit (Stoet, 2017) was used to create a survey that includes a version of the Interpersonal Visual Analog Scale or IVAS (Iachini et al., 2021) that investigates interpersonal distance (IPD) in an analog mode. For the evaluation of IPD participants were presented on the screen of a PC images depicting two different characters on opposite sides of a line: one of the figures (men or women) represented the participant, whereas the other one represented a different subject whose age and gender varied. Participants were asked to move the cursor and indicate the IPD they preferred to maintain from the subject at the opposite end of the line, carrying on the task for all items presented (Figure 1).

### Procedure

Data collection was carried out between July 20, 2021, and October 25, 2021, i.e., following the outbreak provoked by the Delta SARS-CoV-2 variant in Italy. The study involved A.O.U. Policlinico of Bari for the recruitment of participants belonging to the clinical group, the University of Bari “Aldo Moro” for the recruitment of participants belonging to the control group, and the University of Campania Luigi Vanvitelli that provided for the IVAS for the assessment of interpersonal distance (IPD).

COVID-19 patients were included in this study on condition that they were aged between 18 and 65 years and did not suffer from major neurocognitive disorders, based on the Diagnostic and Statistical Manual of Mental Disorders (DSM-5) criteria (American Psychiatric Association, 2013). The exclusion of underlying neurocognitive disorders followed a brief clinical examination and screening.

The clinical group was selected from a pool of subjects scheduled for clinical assessment and medical consultations after having contracted COVID-19. These subjects were assigned to the Unit of Clinical Psychology and Neuropsychology by the referring physicians for either the Department of Internal Medicine or the Department of Occupational Medicine. Thus, well-trained psychologists provided the patients with clinical assessment tools and proper instructions.

All participants were asked to provide written informed consent. Among them, those who met the inclusion criteria for our study could pursue psychological assessment. Sociodemographic data (i.e., age, sex, level of education) were considered in recruiting control group subjects with characteristics identical to those of clinical subjects. Control subjects started an online survey consisting of the same assessment instruments proposed to the clinical group, after having filled in the informed consent through a dedicated online form.

Both clinical and control subjects were administered the psychometric protocol comprising: (1) the Beck Depression Inventory—Second Edition (BDI-II) (Beck et al., 1996); (2) the Impact of Event Scale-Revised (IES-R) (Weiss and Marmar, 1996); (3) the Interpersonal Visual Analog Scale (IVAS) (Iachini et al., 2021). The overall time required by each participant to complete the questionnaires and the task ranged from 15 to 30 min.

### Study Arrangements

The adoption of some precautions allowed to limit potential internal validity bias. In particular, the whole sample shared the same geographical context of origin (Apulian region in Southern Italy), where the same restrictions implemented by the Government to limit the transmission of Sars-Cov-2 were in force. All clinical subjects referred to the U.O.S. of Clinical Psychology and Neuropsychology scheduled for post-COVID monitoring at the General Hospital of Bari were included in the study as they met the preset eligibility criteria. None of them discontinued or withdrew their participation, presenting a response rate of 100%. The same adherence to the assessment procedure was recorded by controls who were paired to clinical subjects based on common sociodemographic characteristics to provide comparability between the two groups and control for potential confounders.

### Statistical Methods

Variance analyses and correlations have been carried out by means of the software package “Statsoft Statistica.” Before carrying out parametric tests, kurtosis and asymmetry tests were performed for all the measurements obtained, suggesting appropriate symmetry and peakedness of the distribution (e.g., DeCarlo, 1997). Two-way ANOVAs were performed to compare the means of the two samples (i.e., Clinical vs. Control; Women vs. Men), on the variables of the study (i.e., depression, post-traumatic symptoms, and IPD). Both gender and diagnosis were included as categorical predictors, whereas the BDI-II, IES-R, and IVAS scores (interpersonal proximity from children, adults, and the elderly) were considered as dependent variables. A repeated-measure ANOVA was used to compare means across variables based on repeated IVAS observations. Bonferroni *post hoc* tests were performed to explore further and to identify which pairs of means are statistically different.

Correlations were performed between the scores at IVAS, BDI-II, and IES-R within the whole group of participants and accounting for the two categories of diagnosis and gender. All correlation findings were corrected through Benjamini–Hochberg False Discovery Rate (FDR) to control for type-I errors when performing multiple comparisons.

## Results

### Sociodemographic and Clinical Variables

A total of 86 participants were recruited for the study. The clinical specimen consisted of 18 men and 25 women aged approximately 50.4 years (SD = 10.1), and with an average education of 13.67 years (SD = 3.99). The control group is composed of women and men in equal numbers (18 men; 25 women) and with the same age (= 50.4; SD = 10.1) and educational level (= 13.67; SD = 3.99) of the clinical group, but who have not contracted the virus.

**FIGURE 2:**
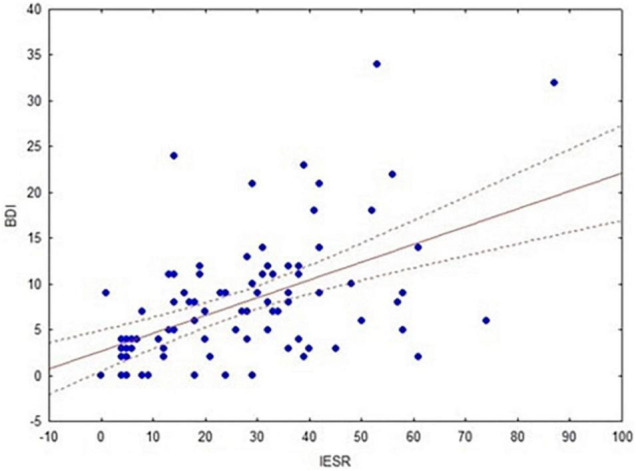
The flow diagram of participants shows the main characteristics of the clinical and control groups.

Table 1 and Figure 2 shows the demographic and clinical characteristics of the participants in the study sample, whereas Table 2 displays BDI-II, IES-R, and IVAS scores reported by both clinical and control subjects.

**TABLE 1:** Sociodemographic variables characterizing the clinical group and the control group.

Variable	Clinical group	Control group
**Age (missing data = 0)**		
Mean (SD)	50.4 (SD = 10.1)	50.4 (SD = 10.1)
Range	26–65	26–65
**Gender (missing data = 0)**		
Men—M	18 (41.86%)	18 (41.86%)
Women–F	25 (58.14%)	25 (58.14%)
**Educational level (missing data = 0)**		
Mean (SD)	13.67 (SD = 3.99)	13.67 (SD = 3.99)
Primary school	1 (2.33%)	1 (2.33%)
Middle school	9 (20.93%)	9 (20.93%)
Secondary school	17 (39.53%)	17 (39.53%)
University degree	16 (37.21%)	16 (37.21%)
Non-contracted COVID-19 (missing data = 43)	–	43
**Contracted COVID-19 (missing data = 43)**		
Non-hospitalized	24 (55.81%)	–
Non-severe	21 (87.5%)	–
Severe	3 (12.5%)	–
Hospitalized	19 (44.19%)	–
Non-severe	9 (47.37%)	–
Severe	10 (52.63%)	–
**Marital status (missing data = 0)**		
Single	5 (11.63%)	6 (13.95%)
Married	33 (76.74%)	37 (86.05%)
Separated/divorced	4 (9.30%)	–
Widowed	1 (2.33%)	–
**Employment status (missing data = 0)**		
Not employed	9 (20.93%)	7 (16.28%)
Employed	34 (79.07%)	36 (83.72%)

**TABLE 2:** Mean scores at BDI-II, IES-R, and IVAS of the clinical group and the control group.

Questionnaire or test	Clinical Group	Control Group
BDI-II	9.53 (SD = 8.17)	6.28 (SD = 4.80)
(Range = 0–63)	0–34	0–23
IES-R	25.35 (SD = 19.13)	28.14 (SD = 17,67)
(Range = 0–88)	1–87	0–74
IVAS		
Child	48.84 (SD = 31.85)	33.66 (SD = 31.75)
(Range = 0–100)	3.83- 100	0–100
Adult	49.00 (SD = 27.32)	36.18 (SD = 28.48)
(Range = 0–100)	0–100	8.5–100
Elderly	50.08 (SD = 30.46)	35.32 (SD = 30.44)
(Range = 0–100)	0–100	0–100

### Analysis of Variance

#### Depressive Symptoms

**FIGURE 3:**
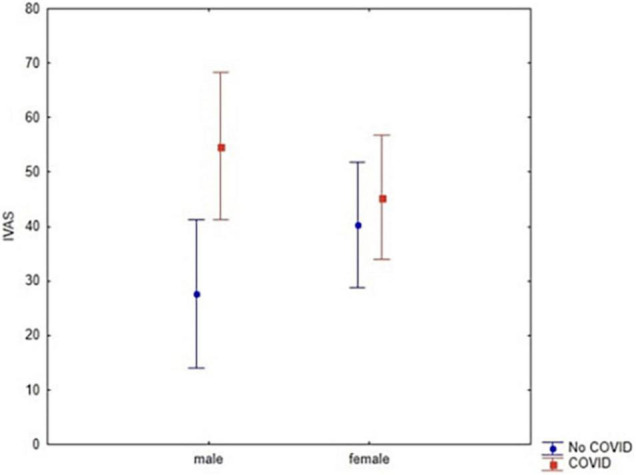
Interaction between Diagnosis and Gender (*F* = 9.08; *p* = 0.003) through BDI-II scores. Vertical bars denote 0.95 confidence intervals.

A 2 × 2 ANOVA with Diagnosis (Clinical vs. Control) and Gender (Women vs. Men) as between-subjects factors, and BDI-II as dependent variable was performed. No Diagnosis effect was detected (df = 1; *F* = 3.5; *p* = 0.06; Cronbach’s alpha = 0.5), although a marginal significant was observed. With respect to gender differences, we found that women had higher BDI-II scores than men (df = 1; *F* = 4.46; *p* = 0.038; Cronbach’s alpha = 0.6). However, a significant interaction between diagnosis and gender on BDI-II scores was observed only among women who contracted COVID-19 (df = 1; *F* = 9.08; *p* = 0.003; Cronbach’s alpha = 0.9; Figure 3). Bonferroni *post hoc* analysis revealed that women who contracted COVID-19 had higher scores than women who did not contract the disease (*p* = 0.002) and men, regardless of their diagnosis (respectively, *p* = 0.003, and *p* = 0.004).

#### The Psychological Impact of Stressful Events

**FIGURE 4:**
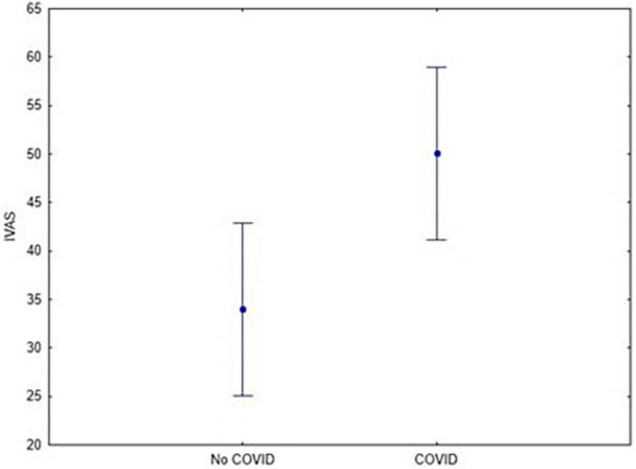
Interaction between Diagnosis and Gender (*F* = 4.63; *p* = 0.03) through IES-R scores. Vertical bars denote 0.95 confidence intervals.

A 2 × 2 ANOVA with Diagnosis (Clinical vs. Control) and Gender (Women vs. Men) as between-subjects factors and IES-R as dependent variable was performed. Results showed a Gender effect, as women reported significantly higher IES-R scores than men (df = 1; *F* = 4.63; *p* = 0.03; Cronbach’s alpha = 0.6). No significant effect emerged for diagnosis (df = 1; *F* = 0.96; *p* = 0.33; Cronbach’s alpha = 0.2) and no significant interaction was found between diagnosis and gender with reference to IES-R scores (df = 1; *F* = 2.64; *p* = 0.11; Cronbach’s alpha = 0.9) (Figure 4).

#### 3.2.3 Interpersonal Distance

**FIGURE 5:**
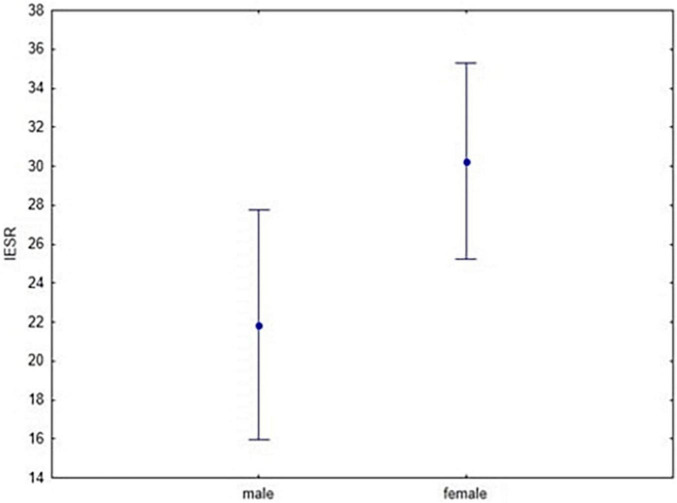
Gender effect on IVAS score (*F* = 6.5; *p* = 0.01). Vertical bars denote 0.95 confidence intervals.

A 2 × 2 mixed ANOVA with Gender (Women vs. Men) as between factor and IVAS scores as within-subjects was performed. We found that Diagnosis may have affected IVAS scores, as participants who contracted COVID-19 had higher scores than those who did not contract the disease (df = 1; *F* = 6.5; *p* = 0.013; Cronbach’s alpha = 0.7, Figure 5).

**FIGURE 6:**
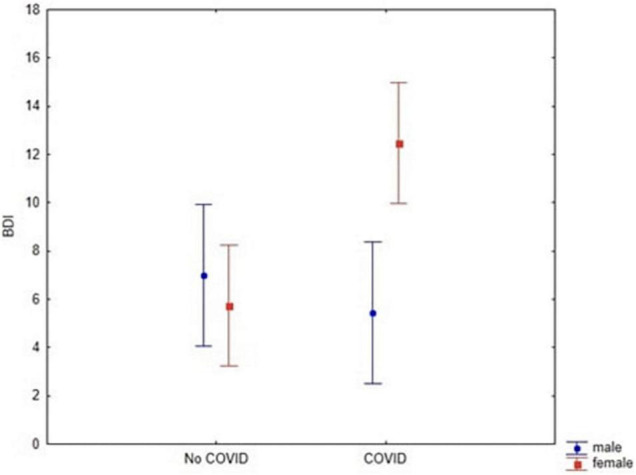
Interaction between Gender and Diagnosis (*F* = 3.05, *p* = 0.08) through IVAS scores. Vertical bars denote 0.95 confidence intervals.

In addition, we found a relevant trend in the interaction between diagnosis and gender (df = 1; *F* = 3.05, *p* = 0.08; Cronbach’s alpha = 0.4; Figure 6) which yet was not significant. As for the other main effects observed through ANOVAs, we further explored this potential interaction through a *post hoc* analysis. Hence, we found that men’s scores varied between the clinical and the control group. In particular, men who did not contract COVID-19 reported lower IVAS scores (Bonferroni *post hoc p* = 0.04). Nevertheless, these data require further investigation to be supported as no (other) significant effect or interaction emerged (all F < 3.05; *p* > 0.08).

The total results of the aforementioned ANOVAs are summarized in Table 3.

**TABLE 3:** Summary table of results obtained from ANOVAs.

Depressive symptoms (BDI-II)	df	df × factor	df -Error	F	p	Cronbach’s alpha
Diagnosis-based differences	3	1	82	3.5	0.06	0.5
Gender-based differences		1	82	4.46	0.038	0.6
Diagnosis and gender		1	82	9.08	0.003	0.9
The psychological impact of stressful events (IES-R)	df	df × factor	df -Error	F	p	Cronbach’s alpha
Diagnosis-based differences	3	1	82	0.96	0.3	0.2
Gender-based differences		1	82	4.63	0.03	0.6
Diagnosis and gender		1	82	2.64	0.11	0.4
Interpersonal distance	df	df × factor	df -Error	F	p	Cronbach’s alpha
Diagnosis-based differences	3	1	82	6.5	0.013	0.7
Diagnosis and gender		1	82	3.05	0.08	0.4

### Pearson’s Correlations

**FIGURE 7:**
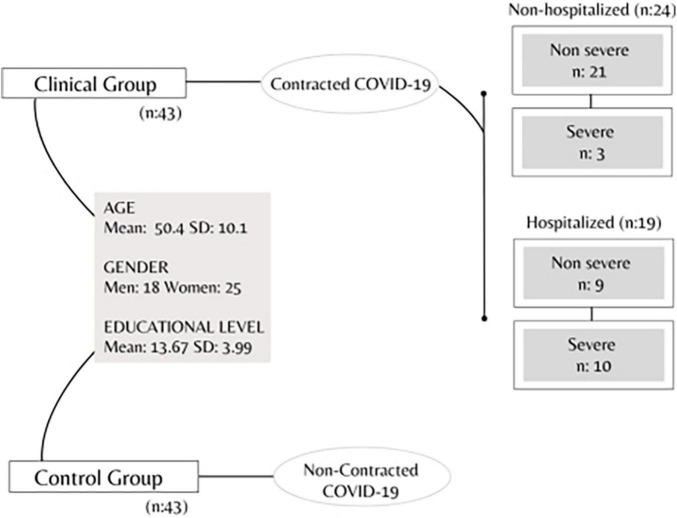
Correlation between BDI-II and IES-R scores within the whole sample.

We found a strong correlation between BDI-II and IES-R scores within the whole sample (*r* = 0.5, *p* = 0.0001, FDR-corrected = 0.0003; Figure 7). The same correlation was observed within the clinical (*r* = 0.65; *p* = 0.0001, FDR-corrected = 0.0003) and the control group (*r* = 0.41; *p* = 0.006, FDR-corrected = 0.009), respectively. Another significant correlation concerned the BDI-II and IES-R scores within women (*r* = 0.51; *p* = 0.0001, FDR-corrected = 0.0003) and men (*r* = 0.48; *p* = 0.003, FDR-corrected = 0.006), respectively.

**FIGURE 8:**
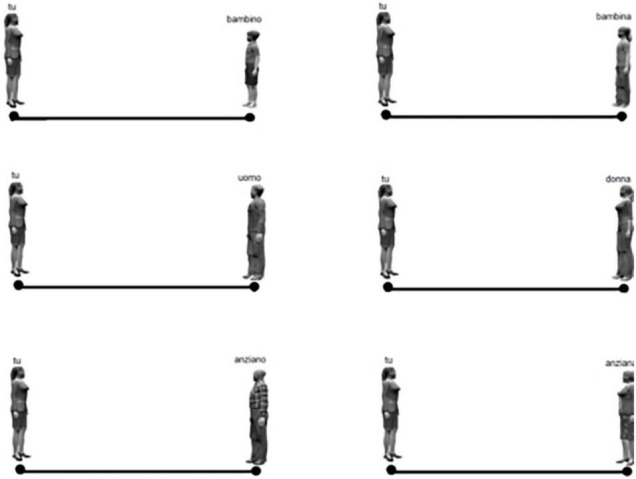
Correlation between IVAS and IES-R scores within the women group.

Additionally, we performed a Pearson’s correlation between IVAS scores, BDI-II, and IES-R. No significant associations between BDI-II and IVAS (*r* = −0.05; *p* = 0.66, FDR-corrected = 0.66), and IES-R and IVAS (*r* = 0.08; *p* = 0.47, FDR-corrected = 0.5) emerged within the whole sample. Conversely, a relevant trend pertaining to the correlation between IVAS and IES-R scores was found within the women group (*r* = 0.29; *p* = 0.04, FDR-corrected = 0.056; Figure 8). No other significant correlation emerged within the men group, regardless of the COVID-19 diagnosis (all r < 0.05; *p* > 0.7).

## Discussion

The present research work sought to examine changes in mood and distress based on having or not contracted the COVID-19, and how changes in these psychological variables may have been reflected in the perception of interpersonal distance. The study also investigated gender differences, revealing that women might have experienced a greater psychological burden than men and they are also more prone to experience severe depressive and post-traumatic symptoms. In particular, women who contracted the disease showed higher levels of depression than men. The analysis of interpersonal distance revealed people who contracted the infection tend to prefer greater distancing from others. Further associations between depressive and post-traumatic symptoms as well as potential salient trends between post-traumatic symptoms and IPD were observed within the women group.

The findings of our study thus indicated that the women may be related to a higher occurrence of depression and probable PTSD. Depressive symptoms in women also seem to be associated with having contracted COVID-19 as women who contracted the disease reported higher scores at the BDI scale measuring depressive state. These results appear to be in line with previous research that highlighted the association between the women and increased risks for psychological discomfort (Mazza C. et al., 2020; Qiu et al., 2020; Wang et al., 2020), as well as evidence of women’s vulnerability to distress and the development of symptoms compatible with PTSD (Sareen et al., 2013; Liu et al., 2020). These findings also appear consistent with the literature concerning women’s exposure and risks for psychological distress (Sareen et al., 2013; Liu et al., 2020; Mazza M. G. et al., 2020; Qiu et al., 2020; Wang et al., 2020).

Furthermore, this study showed how having contracted COVID-19 may have affected interpersonal distance, made manifest by the propensity of participants who have suffered from the disease to maintain greater distances from adults, children, and the elderly, compared to those who have never been infected. The increase in interpersonal proximity has also been evaluated in previous studies as a useful method for dealing with epidemic situations (Caley et al., 2008). To date, current recommendations suggest standing about 2 meters away from others while wearing protective facial masks, whereas without the masks an even greater IPD is expected (Chu et al., 2020; Setti et al., 2020). The maintenance of the recommended long distances (i.e., social distance) represents an ongoing challenge because it would result in a change in the natural proxemic preference that is less than one meter only in informal meetings (Welsch et al., 2020), although the perception of interpersonal space has undergone a strong variation due to the pandemic (Iachini et al., 2021). The analyses also revealed a tendency for men to maintain less interpersonal distance from children, adults, and the elderly. This behavior does not appear to be related to disease contraction and is consistent with the literature stating that, in general, the women have higher risk perceptions than men and tend to estimate a lower likelihood of the COVID-19 crisis being solved completely, leading to a return to “normal,” everyday life.

Moreover, recent COVID data showed that women are more concerned about COVID-19 than men (Gerhold, 2020) that may result in behavioral changes because of their beliefs and expectations related to the pandemic event (Alsharawy et al., 2021). Consequently, our data may be consistent with an increase in women’s perception of distress that may be reflected through the adoption of greater proximal behavior than men. also considering the high rate of comorbidity between PTSD and major depressive disorder (Elhai et al., 2008; Roley et al., 2015). During the COVID-19 pandemic, several symptoms attributable to PTSD have emerged as intense and persistent even after recovery in individuals with a history of depression who had previously contracted the virus (Einvik et al., 2021).

In our study, emerged a positive correlation between scores indicative of probable post-traumatic stress disorder and greater mood deflection was indicated by scores to BDI-II and IES-R. These findings also revealed gender-related differences with a greater tendency for women to obtain higher scores, consistent with the literature (Galasso et al., 2020; Alsharawy et al., 2021).

Finally, findings obtained from correlational analyses may lay the groundwork for further investigation into correlations between scores indicative of probable post-traumatic stress disorder and greater interpersonal distance. Indeed, despite the lack of data on long-term adaptation strategies for IPD in response to the COVID-19 health crisis, we know that traumatic situations can persistently increase individuals’ need for greater IPD (Bogovic et al., 2014). Such eventuality could also occur during and after the current pandemic that it has been defined as an impactful, trauma-related event (Forte et al., 2020; González-Sanguino et al., 2020; Johnson et al., 2020; Liu et al., 2020). Overall, the preliminary evidence obtained may also set the stage for new data collection and analysis to gain a better understanding of the potential interactions between diagnosis and gender in IPD adjustment, besides the associations between post-traumatic symptoms and IPD according to gender.

### Limitations

Although our results are referred to subjects residing in Southern Italy, risk perception and concern about contagion was found to be high across the country (Lanciano et al., 2020), consistent with the WHO statement about the global impact of the pandemic (Alsharawy et al., 2020). The relatively small sample size reflects, however, the need to collect further data from additional participants and at other stages in the progression of COVID-19 transmission, including the randomized use of digital versions of the psychometric measures used. Albeit significant, these preliminary findings may indeed have limited clinical translatability in improving understanding of COVID-19 from both psychological and psychosocial perspectives. Moreover, the estimates deriving from the administration of both online and offline assessment protocols, as well as from the virtual task for interpersonal proximity may not be generalizable to a varied array of real-life contexts, where other external variables might affect the actual spatial and social behavior.

Future studies with a larger sample should account for potential predictors of depression, post-traumatic symptoms, and variation in IPD preference within the clinical group (e.g., considering the type, severity, and persistence of COVID-19 symptoms; ICU stay; oxygen supply; timepoint of COVID-19 diagnosis, negativization, hospital discharge, and/or complete recovery; duration of hospitalization). In particular, based on routine hospital care, it would be advisable to explore, in more detail, if there could be differences within the clinical group between patients who were hospitalized and those non-hospitalized, and between individuals who suffered from a non-severe disease and those who contracted COVID-19 in a severe form as. Further, the investigation of the role played by key factors such as, for instance, resilience, overall quality of life, burden of isolation measures, and worries about contracting the disease, besides contact history and risk factors for severe disease, may be warranted.

## Conclusive Remarks

The present study involved 86 participants to appraise differences by diagnosis and gender related to the presence of depressive, post-traumatic symptoms, and proxemic preferences following the peak of Sars-Cov-2 Delta variant in Italy. Psychological assessment relied on the combined use of direct (self-report) and indirect (visual analogic) measures to delve into both psychological distress and distance behavior related to COVID-19, also considering differential gender vulnerability to the psychological repercussions of the disease on psychosocial functioning. On the whole, these preliminary findings could contribute to a better understanding of the psychological and psychosocial correlates and potential implications of COVID-19 on both disease-experienced and healthy individuals, setting the basis for future research and the design of more targeted interventions to foster mental health during and after the pandemic.

## Data Availability Statement

The datasets presented in this study can be found in online repositories. The names of the repository/repositories and accession number(s) can be found below: Forms, material, and data are available on the Open Science Framework (OSF): https://osf.io/qtvwe/.

## Ethics Statement

The study was given ethical approval by the Interregional Ethics Committee of the “Azienda Policlinico” of Bari, and executed according to the Declaration of Helsinki (No. ET-20-01). The patients/participants provided their written informed consent to participate in this study.

## Author Contributions

PT: concept and design of the work, acquisition of the data, development of the analysis and interpretation of data, and drafting the manuscript. TL, FA, FB, and CA: concept and the design of the work, acquisition and interpretation of data, and manuscript revision. AM, FS, SC, JL, and GR: acquisition and interpretation of the data. AS, SI, LV, AV, MD, and AC: concept and the design of the work, analysis and interpretation of data, and critical revision. All authors contributed to the article and approved the submitted version.

## Conflict of Interest

The authors declare that the research was conducted in the absence of any commercial or financial relationships that could be construed as a potential conflict of interest.

## Publisher’s Note

All claims expressed in this article are solely those of the authors and do not necessarily represent those of their affiliated organizations, or those of the publisher, the editors and the reviewers. Any product that may be evaluated in this article, or claim that may be made by its manufacturer, is not guaranteed or endorsed by the publisher.
